# On-chip spatiotemporal electrophysiological analysis of human stem cell derived cardiomyocytes enables quantitative assessment of proarrhythmia in drug development

**DOI:** 10.1038/s41598-018-32921-1

**Published:** 2018-09-28

**Authors:** Yumiko Asahi, Tomoyo Hamada, Akihiro Hattori, Kenji Matsuura, Masao Odaka, Fumimasa Nomura, Tomoyuki Kaneko, Yasuyuki Abe, Kiyoshi Takasuna, Atsushi Sanbuissho, Kenji Yasuda

**Affiliations:** 1 Medicinal Safety Research Laboratories, Kasai R&D Center, Daiichi-Sankyo Co. Ltd., Edogawa, Tokyo, 134-8630 Japan; 2 Institute of Biomaterials and Bioengineering, Tokyo Medical and Dental University, 2-3-10 Kanda-Surugadai, Chiyoda, Tokyo, 101-0062 Japan; 3 Organization for University Research Initiatives, Waseda University, 3-14-9 Ookubo, Shinjuku, Tokyo, 169-0072 Japan; 4 Waseda Bioscience Research Institute in Singapore (WABOIS), Helios, 11 Biopolis Way, 138667 Singapore; 5 Department of Pure and Applied Physics, Graduate School of Advanced Science and Engineering, Waseda University, 3-4-1 Okubo, Shinjuku, Tokyo, 169-8555 Japan; 6 Department of Physics, School of Advanced Science and Engineering, Waseda University, 3-4-1 Okubo, Shinjuku, Tokyo, 169-8555 Japan; 7 Present Address: Chiome Bioscience Inc. Shibuya, Tokyo, 151-0071 Japan; 8 Present Address: Department of Frontier Bioscience, Hosei Univ., Koganei, Tokyo, 184-8584 Japan

## Abstract

We examined a simultaneous combined spatiotemporal field potential duration (FPD) and cell-to-cell conduction time (CT) in lined-up shaped human embryonic stem cell-derived cardiomyocytes (hESC-CMs) using an on-chip multielectrode array (MEA) system to evaluate two origins of lethal arrhythmia, repolarization and depolarization. The repolarization index, FPD, was prolonged by E-4031 and astemizole, and shortened by verapamil, flecainide and terfenadine at 10 times higher than therapeutic plasma concentrations of each drug, but it did not change after lidocaine treatment up to 100 *μ*M. CT was increased by astemizol, flecainide, terfenadine, and lidocaine at equivalent concentrations of Nav1.5 IC_50_, suggesting that CT may be an index of cardiac depolarization because the increase in CT (*i*.*e*., decrease in cell-to-cell conduction speed) was relevant to Nav1.5 inhibition. Fluctuations (short-term variability; STV) of FPD and CT, STV_FPD_ and STV_CT_ also discriminated between torsadogenic and non-torsadogenic compounds with significant increases in their fluctuation values, enabling precise prediction of arrhythmogenic risk as potential new indices.

## Introduction

Lethal arrhythmias, including torsades de pointes (TdP) and ventricular tachycardia (VT), are a critical safety issue in drug development. To exclude torsadogenic compounds, the International Council for Harmonisation of Technical Requirements for Pharmaceuticals for Human Use (ICH) has implemented the two essential assays, *in vitro* human ether-a-go-go-related gene (hERG) and *in vivo* QT assays, and additional *in vitro* action potential duration (APD) as a follow up under the ICH S7B guideline^1^. However, *in vitro* hERG, *in vitro* APD and *in vivo* QT assays still have difficulty in fully predicting lethal arrhythmias, resulting in some compounds are judged as false negative or false positive^1–3^ in these assays.

Human pluripotent stem cell-derived cardiomyocytes (hPSC-CMs), which expresses physiologically functioning ion channels, have been developed as a more appropriate cell source for assessing proarrhythmia risks. Especially in the multi-electrode array (MEA) assay, extracellular recording of field potential duration (FPD) prolongation, which is equivalent to APD and QT interval prolongation, can predict clinical QT prolongation and/or arrhythmogenic liability more accurately than existing *in vitro* and *ex vivo* assays^4–6^. In a typical waveform obtained from field potential recordings of hPSC-CMs, FPD is defined as the temporal interval from the first peak of the fast, sharp wave component to the second peak in the slow, broad wave component, and the duration is mostly reflected by I_*Kr*_ along with other cardiac ionic currents such as I_*Na*_, I_*Ca*_, and I_*Ks*_. However, FPD prolongation also cannot fully predict lethal arrhythmia or QT prolongation, particularly for arrhythmias induced by multi-channel effects.

To improve the clinical relevance, various efforts have been made in the MEA assay using hPSC-CMs. For example, it is possible to analyze waveform abnormalities, such as early after depolarization, triggered activity and ectopic beats, which are potent proarrhythmia markers for both I_*Kr*_ inhibitors and multi-channel blockers^7,8^. Moreover, the combination with FPD in MEA assays and other types of assays, such as impedance, motion field imaging, Ca^2 +^ transient, beating pattern assessment, and *in silico* simulation based on multi-ion channel activities, improves prediction of cardiac liabilities and provides insight into the mechanism-of-action of drugs^6,9–13^. It has been reported that intracellular recording of APD in hESC-CMs shows overall pharmacological sensitivity and predictability of the cardiac risk of arrhythmogenic drugs^14^. Furthermore, interestingly, the temporal fluctuation, that is, short-term variability (STV) of APD in hESC-CMs, has specificity that recognized moxifloxacin as a safe drug, although APD itself was prolonged^15^. The STV of APD or STV of QT has been shown to identify individuals at high risk of arrhythmia *in vivo* and in clinical^16–18^. In addition, this approach would be also proposed to be useful for precision medicine but not only for cardiotoxicity assessment by using patient-derived hPSC-CMs^19,20^. These findings suggest that a surrogate arrhythmic marker such as STV, which is not exclusively dependent on hERG inhibition or QT prolongation, is needed for appropriate judgment of the arrhythmnogenic risk of drugs. STV of the QT interval or APD, which represent a temporal fluctuation, has been well studied and known as a quantitative proarrhythmic marker in *in vivo* animal models, isolated primary cardiomyocytes, and retrospective analysis of clinical observation^21–25^. However, little is currently known about influence on temporal fluctuation of FPD in the MEA assay using hPSC-CMs (*i*.*e*. STVs of FPD).

The ion channel panel assay has been proposed, consisting of six ion channels (I_*Kr*_, I_*Na*_, I_*Ca*_, I_*Ks*_, I_*to*_ and I_*K*1_), whose currents are important in not only repolarization but also depolarization of the cardiac action potential^26^. From the viewpoint of cell-to cell conduction of cardiomyocytes, sodium channel blockers have been well studied in both *in vitro* cardiomyocytes and *in vivo* animal models. It has been reported that quinidine and flecainide decelerate the electrical conduction, although lidocaine or mexiletine show no or a lower effect on conduction in animal-isolated cardiomyocytes^27,28^. Clinically, proarrhythmia induced by sodium channel blockers is limited to class Ia (*e*.*g*., quinidine) and class Ic (*e*.*g*., flecainide) agents^29,30^, whereas class Ib agents (*e*.*g*., lidocaine and mexiletine) appear to be safe^31,32^. In addition, not only deceleration of conduction, but also spatiotemporal fluctuation in both cell-to cell conduction and APD can lead to VT and ventricular fibrillation (VF)^33–36^. However, there are few reports focusing on the relationship between depolarization delay and slowing of cell-to cell conduction.

We have described improvement of the synchronous beating behavior of cardiomyocytes with an increase in their cell number in clusters as a “community effect”^37,38^. Using an on-chip constructive approach, interbeat intervals (IBIs) of two neighboring isolated cardiomyocytes synchronize with the IBI of the more stable cardiomyocyte (i.e., lower coefficient of variability (CV) of IBI) regardless of their IBI speed^39^. These results indicated the importance of cell-to-cell connection in the *in vitro* cardiomyocyte screening assay because the response might be ruled by the most stable cardiomyocytes in the inhomogeneous cardiomyocyte clusters. Hence, we have previously demonstrated that lined-up hESC-CMs in the MEA assay can be an *in vitro* small-scale model to detect cell-to cell conduction and its STV (spatiotemporal fluctuation)^40^. Cell-to cell conduction was evaluated by conduction time (CT) between two neighboring electrodes with 150 *μ*m inter-electrode distance in the lined-up cell network using hESC-CMs. In this report, we examined effects of E-4031 and verapamil, well known drugs with QT-prolongation and -shortening effect, respectively, on FPD, CT, and their STVs in lined-up hESC-CMs. E-4031 had a QT-prolonging effect and verapamil showed a QT-shortening effect. Furthermore, we investigated the effects of sodium channel blockers (flecainide and lidocaine,; positive and negative control for cell-to cell conduction, respectively) and two other arrhythmogenic drugs as multi-channel blockers (astemizole and terfenadine) on these four parameters.

This is the first report evaluating spatiotemporal fluctuation of CT and FPD using diverse ion channel blockers in the MEA assay employing hESC-CMs. The quantitative multi-parametric analysis may indicate more precise cardiac ion channel blocker-induced cardiac liability.

## Principle

The extracellular field potential(FP) is the electrical potential produced by the inward and outward ion currents of cardiomyocytes. In electrophysiological conventions, a negative current value or downward deflection of a current trace are typically referred to as an inward current, reflecting either the movement of positive ions into the cell or negative ions out of the cell. A positive current value or upward deflection of the current trace (*i*.*e*., outward current) also reflect either the movement of positive ions out of the cell or negative ions into the cell. Similar to the action potential(AP), the FP origin is the electrical potential difference across the plasma membrane of excitable cells caused by the inward/outward ion current of cells. Hence, for individual cells, the time course of FP, *V*_*f*_, is proportional to the transmembrane ion current of cells, *I*_*ion*_, which is the AP time differential, *ϕ*_*a*_, of cells. The relationship of *V*_*f*_ to *ϕ*_*a*_ is theoretically described as,

1 $${V}_{f}={I}_{ion}R=R\frac{d{\varphi }_{a}}{dt}$$

where, *R* is the resistance of the environment and measurement system. Time course *V*_*f*_ amplitude is the value of the FP microelectrode measurement.

**Figure 1:**
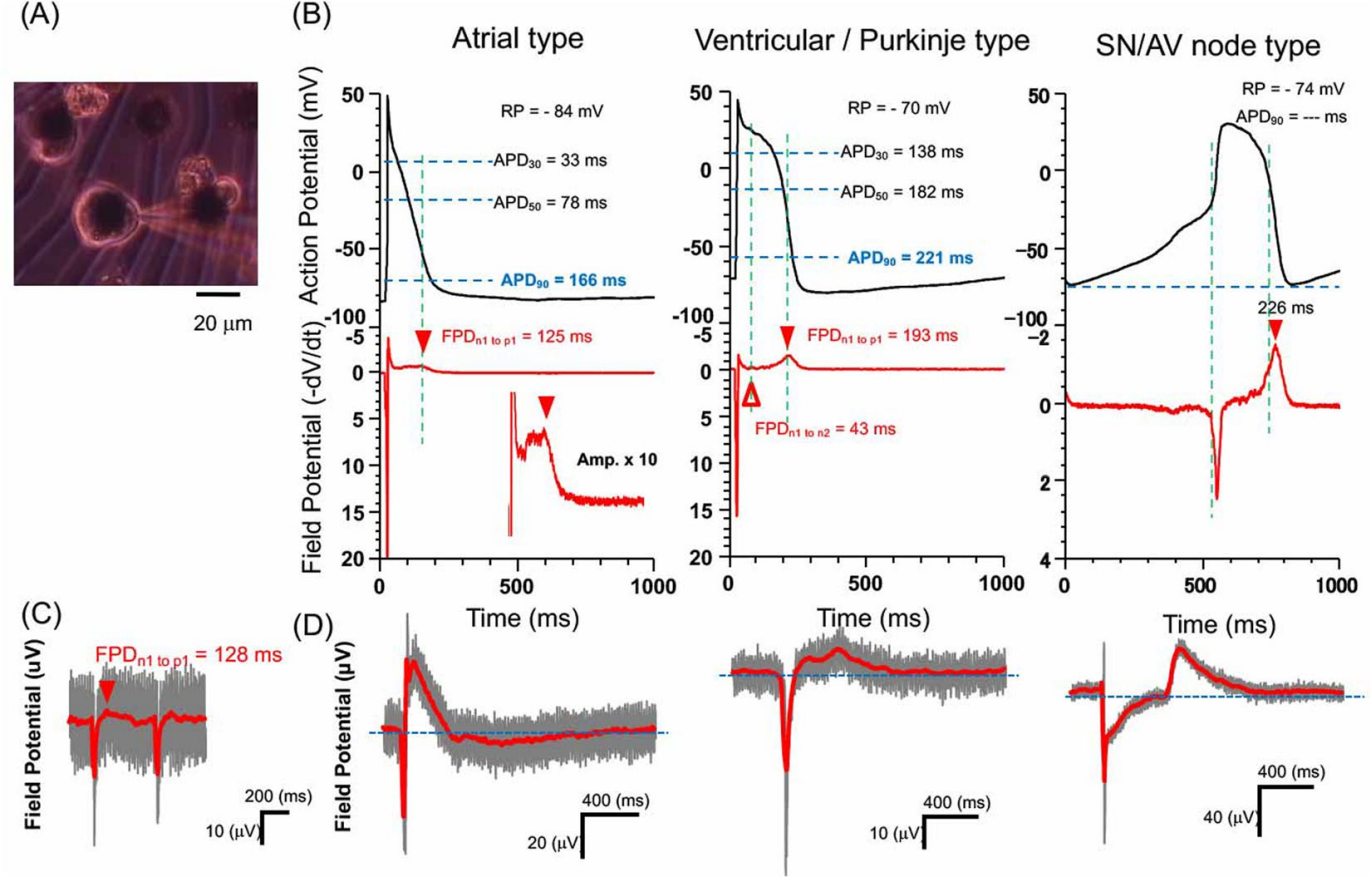
Relationship of the external field potential and action potential of cells. (**A**) Micrograph shows an example of electrophysiological measurement set-up of external field potential (FP: with 10 *μ*m multi electrode array) and action potential (AP: patch clamp) of single cardiomyocytes. (**B**) Examples of action potentials of three types of single cardiomyocytes (atrial (left), ventricle (center), sinus node (right)), and their time differential plots (−dV/dt). (**C**) Typical FP signal of a single cardiomyocyte on a 10 *μ*m microelectrode. (**D**) Examples of FPs of three types of cardiomyocytes.

Because phenotypes and electrophysiological changes of cardiomyocytes can be distinguished by APs, in practice, FPs can be used for long term screening of the electrophysiological properties of cells on microelectrodes. The actual field potential waveform measured by MEA is affected by the electrical resistance and capacitance between the cell membrane and the surface of electrode^41^, however, as shown in Fig. 1, FP waveforms of phenotypes can be estimated by the time differential of APs, and the experimental results of FPs were similar to their time differentials of APs.

## Materials and Methods

### Test compounds

The following six compounds in four categories were chosen and applied for experiments: E-4031; hERG inhibitor, astemizole and terfenadine; multi-channel blockers, lidocaine and flecainide; sodium channel blocker, and verapamil; calcium channel blocker. The vehicle control was dimethyl sulfoxide (DMSO). E-4031 was synthesized in Daiichi Sankyo Co., Ltd (Tokyo, Japan). The other compounds were purchased from Sigma (St. Louis, MO). Each compound was dissolved in DMSO at a thousand-fold concentration to prepare stock solutions. The highest concentration of each compound was 30 100 times their effective therapeutic plasma concentration (C_*eff*_) in consideration of the solubility.

### Cell culture

Human embryonic stem cell-derived cardiomyocytes clusters (hES-CMCs) were shipped from Cellectis (hES-CMCTM002, hES cell line SA002, Gothenburg, Sweden)^42,43^, and maintained in Dulbecco’s modified Eagle’s medium (DMEM) supplemented 20% heat-inactivated fetal bovaine serum, 1 mM GlutaMax, 100 U/mL penicillin, 0.1 mg/mL streptomycin, 1% nonessential amino acids and 0.1 mM *β*-mercaptoethanol (Invitrogen, Carsbad, CA, USA). Dispersed cardiomyocytes were isolated from hES-CMCs by a modification of the methods of Norström *et al*.^44–48^. Briefly, 30 to 90 clusters were treated with 0.25% trypsin-EDTA (Invitrogen) added DNase for 2–3 min at 37 °C in 5% CO_2_, and then, were centrifuged at 200 × g for 2–3 min at 4 °C. The supernatant was gently aspirated with pipette, and a 5-hold volume of DMEM was added as stop solution. Remaining clusters were treats ed with the above process with several times. The supernatants were collected and centrifuged with 200 × g for 2–3 min at 4 °C. The resulting supernatant including dispersed cells was seeded into agarose microchambers on an MEA chip (MED-P530A; AlphaMED scientific. Co. Ltd., Japan)^40^.

### MEA cardiomyocyte lined-up network chip

**Figure 2:**
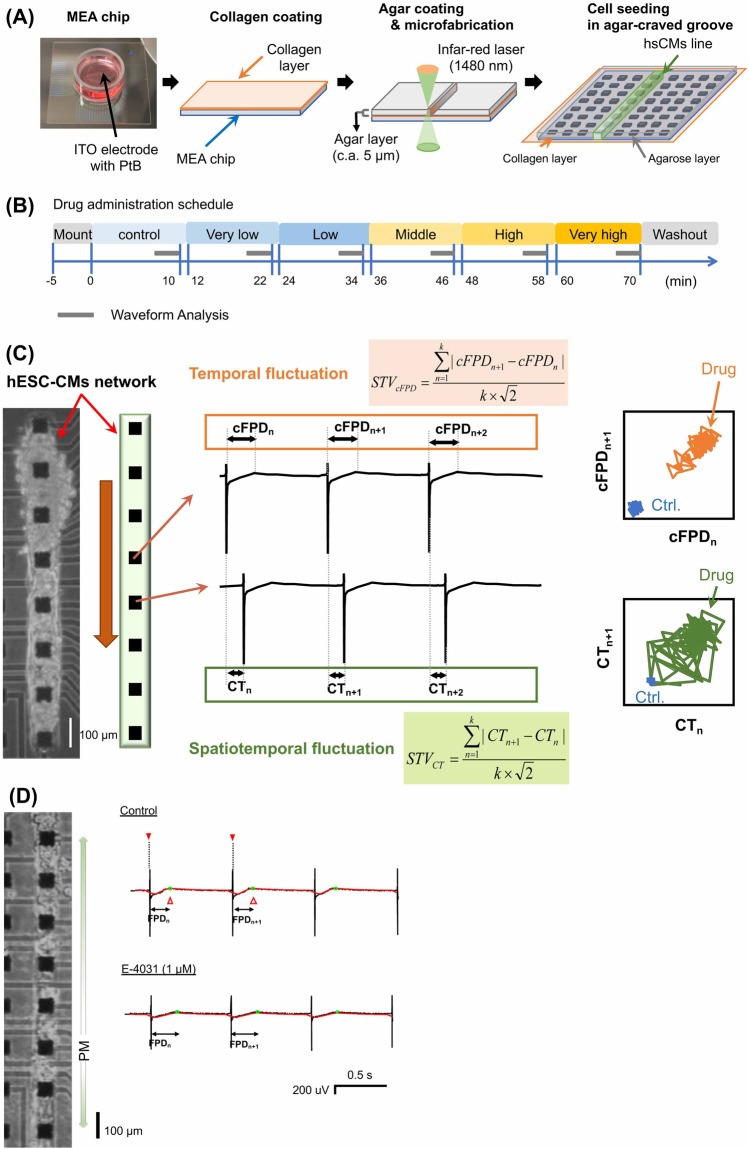
Experimental designs of temporal and spatial fluctuation measurement. (**A**) Preparation of linearly-craved MEA chip with collagen coating. A schematic drawing of MEA chip for the lined-up hESC-CMs. (**B**) Time-course of cell culture and drug administration. FP waveforms were recorded during 10 min of drug exposure, and last 50 waveforms of obtained data were analyzed to calculate cFPD, STV_*cFPD*_, CT and STV_*CT*_. (**C**) Assay parameters measured in the multi-electrode array (MEA) system. cFPD: field potential duration corrected by Bazett’s formula, CT: conduction time which is time difference of first peak from that of an adjacent channel, STV_*cFPD*_: Short term variability of cFPD. The image of fluctuations of cFPD and CT was shown as Poincaré plottings. (**D**) Representative change in lined-up hESC-CMs with administration of E-4031 (hERG blocker).

The MEA chip surface was prepared as follows. First, the chip surface was coated with collagen type I-C (Nitta Gelatin, Japan). After drying, the chip surface was coated with a 0.1% agarose gel. A part of thin agarose layer was carefully melted along three parallel lines of 8 × 8 multi-electrodes with spot heating of infra-red laser (1480 nm), which selectively removed agarose gel, but not collagen gel (Fig. 2). At >30 min before the start of experiment, the medium was changed to 2 mL pre-warmed fresh maintenance medium per chip.

### Field potential recordings

Extracellular potential recordings of the lined-up hESC-CMs were performed using the MEA system at a sampling rate of 10 kHz with a low path filter of 2 kHz and high path filter of 1 Hz, and signals were amplified by 100–50,000 using the amplifier. The MEA chip with lined-up hESC-CMs was set in the FP measurement device and placed in a humidified cell culture incubator at 37 °C, 5% CO_2_. The signals were analyzed by Igor Pro 6.0 (WaveMetrics, Inc., USA) and FlexPro 7.0 (Weisang GmbH, Germany).

### Drug administration protocol

We adopted the same drug administration protocol as we examined previously^40^ (Fig. 2B). Briefly, the MEA chips of lined-up cells for experiments were selected from the chips having 0.6–1.1 Hz beating frequency and ventricle-like waveforms of FP recordings (see Fig. 1B,D). The MEA chip was placed in the holder of the FP measurement device, and equilibrated for 5 min, and then the control FP waveforms were recorded for 10 min. Subsequently, the drug was applied to the medium at 1% dilution in serially increasing additions, and the FP waveforms were recorded for 10 min at each concentration. Finally, the drug-containing medium was replaced with fresh medium after washing three times. The last 50 beats extracted from 10 min recorded FP waveform data was used for beat rate, FPD (the time from first depolarization peak to broad repolarization peak) and CT (the time difference of the first peaks between separately placed electrodes in lined-upped cell-network) measurement at each concentration (Fig. 2C).

### Data analysis

**Figure 3:**
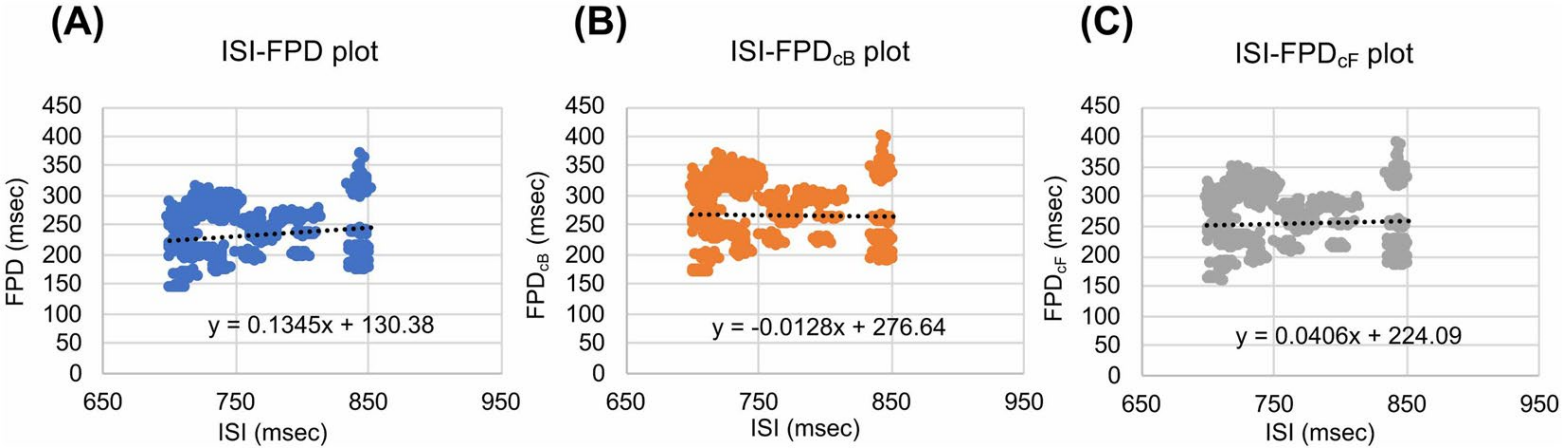
Relationship of FPD or cFPD and inter-spike interval (ISI). Scatter plots of ISI and FPD (**A**), cFPD corrected by Bazett’s formulae (FPD_*cB*_) (**B**), and cFPD corrected by Fridericia’s formulae (FPD_*cF*_) (**C**) in line-up hESC-CMs. All data from vehicle control was plotted. Bazett’s and Fridericia’s formulae for correcting FPD are given as FPD/(ISI)^1/2^ and FPD/(RR)^1/3^, respectively.

The FPD was defined as the duration time between the initial field potential deflection and the peak of inward current of depolarization mainly caused by potassium ion channels. It was normalized (cFPD) to the beating rate of cardiomyocytes using Bazett’s correction formula based on the comparison of the slope of a plot of inter-spike interval and FPD, or corrected FPD by Bazett’s or Fridericia’s formulae (Fig. 3). The short-term variability (STV) of cFPD and CT, which was defined as the mean distance of points perpendicular to the line of identity in the Poincaré plot, was calculated ($STV=\sum \,\tfrac{|{D}_{(n+1)}-{D}_{n}|}{(n\times \sqrt{2})}$, where *D*_*n*_ represents the FPD and CT of n-th beating). STVs were last 50 beat data from 5 min extraction of cFPD and CT. To assess the arrhythmogenicity risk of test drugs, the mean values of cFPD, STV_*cFPD*_, CT and STV_*CT*_ of each drug were plotted. Vehicle, verapamil and lidocaine were recognized as safe drugs, and E-4031, astemizole, terfenadine and flecainide were recognized as the arrhythmogenic drugs. In CT-STV_*CT*_ and STV_*cFPD*_-STV_*CT*_ plots, the line segregates the safe and arrhythmogenic drugs. The slope and intercept of the line were determined as the maximum distances from the line to the nearest point in each safe and arrhythmogenic drug data by Microsoft^©^ Office Excel 2013 solver.

### Statistical analysis

All values are presented as mean ± S.E.M. (unless stated otherwise). All drug effects were evaluated using the Dunnett’s multiple comparison test when comparing multiple groups with time-matched vehicle control. P < 0.05 was considered as statistically significant.

## Results

### The effect on cFPD and STV_*cFPD*_

To evaluate the potential to detect the effects on FPD of the lined-up cardiomyocytes network, ca. 1000 hESC-CMs were seeded on the rectangular agarose-free region of the MEA chip and incubated until the cells attached to the microelectrodes efficiently enough to acquire their field potential signals (Fig. 2C). As shown in the field potential graphs, waveforms of hESC-CMs in this experiment were similar to the ventricle type (Fig. 1B).

**Figure 4:**
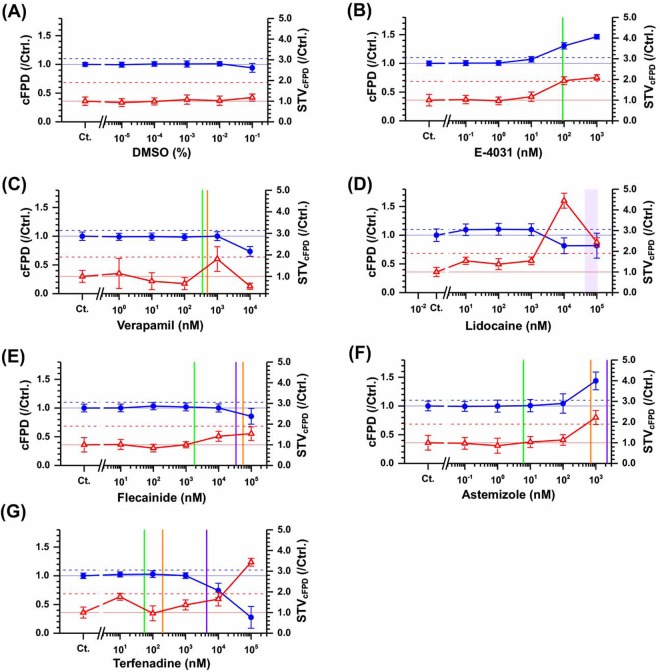
Effects of the arrhythmic drugs to the repolarization function and its temporal fluctuation on the line-shaped model using hESC-CMs. Dose-dependent changes of the field potential duration corrected by beating frequency using Bazett’s formula (cFPD, blue filled circles and lines) and its short-term variability (STV_*cFPD*_, red open triangles and lines) are plotted on DMSO as a vehicle control (**A**), E-4031 as a I_*Kr*_ blocker (**B**), verapamil as a Cav1.2 blocker (**C**), lidocine and flecainide as I_*Na*_ blockers (**D** and **E**), astemizole and terfenadine as multi-ion channel blockers (**F** and **G**). cFPD and STV_*cFPD*_ were normalized to control condition. Symbols and error bars indicate mean values and standerd error (SE). 10% cFPD prolongation (cFPD > 1.1) and 90% STV_*cFPD*_ increase (STV_*cFPD*_ > 1.9) are shown in blue or red dashed lines, respectively. The green, orange, and purple bars show hERG, Cav1.2, and Nav1.5 IC_50_ of each drug, respectively.

We first tested QT-prolonging drug E-4031 with hERG channel blocking as positive control. E-4301 prolonged and was 30% in corrected FPD with Bazett’s formula (cFPD)(n = 13, P < 0.05, compared to DMSO by Dunnett’s Multiple Comparison Tests) at 0.1 *μ*M and 47% (n = 12, P < 0.05) at 1 *μ*M (blue symbols in Fig. 4A,B). Verapamil, calcium channel blocker with an almost equivalent hERG blocking action, shortened cFPD by 26.6% at 10 *μ*M (n = 3, P < 0.05, Fig. reff3C). STV of cFPD (STV_*cFPD*_), which is the fluctuation of the repolarization of cells on the electrode, was more than doubled by 1 *μ*M of E-4031, whereas no significant change in STV_*cFPD*_ was observed at concentrations up to 10 *μ*M of verapamil (Fig. 4B,C, red symbols). Responses to cFPD in this lined-up hESC-CMs and cluster were similar^40^.

Next, we tested lidocaine, flecainide, astemizole and terfenadine. Lidocaine, of which Nav1.5 IC_50_ is 42–108 *μ*M, shortened cFPD at 10–100 *μ*M (−18%, P < 0.05, Fig. 4D). Flecainide, for which Nav1.5 IC_50_ is approximately 34 *μ*M with hERG IC_50_ at 1.8 *μ*M and Cav1.2 IC_50_ at 56 *μ*M, shortened cFPD at 100 *μ*M (−14%, P < 0.05, Fig. 4E). Astemizole prolonged cFPD by 43.7% at 1 *μ*M (n = 3, P < 0.05, Fig. 4F), but terfenadine shortened cFPD at 10 or 100 *μ*M (−26%, P < 0.05, n = 4, Fig. 4G or −78%, n = 2). STV_*cFPD*_ was increased by 1 and 10 *μ*M lidocaine (345 and 143%, P < 0.05, Fig. 4D) as well as 1 *μ*M of astemizole (122%, P < 0.05, Fig. 2F) and 100 *μ*M of terfenadine (243%, P < 0.05, Fig. 4G), while it was increased about 50% by 10–100 *μ*M flecainide (Fig. 4E).

### The effect on CT and STV_*CT*_

**Figure 5:**
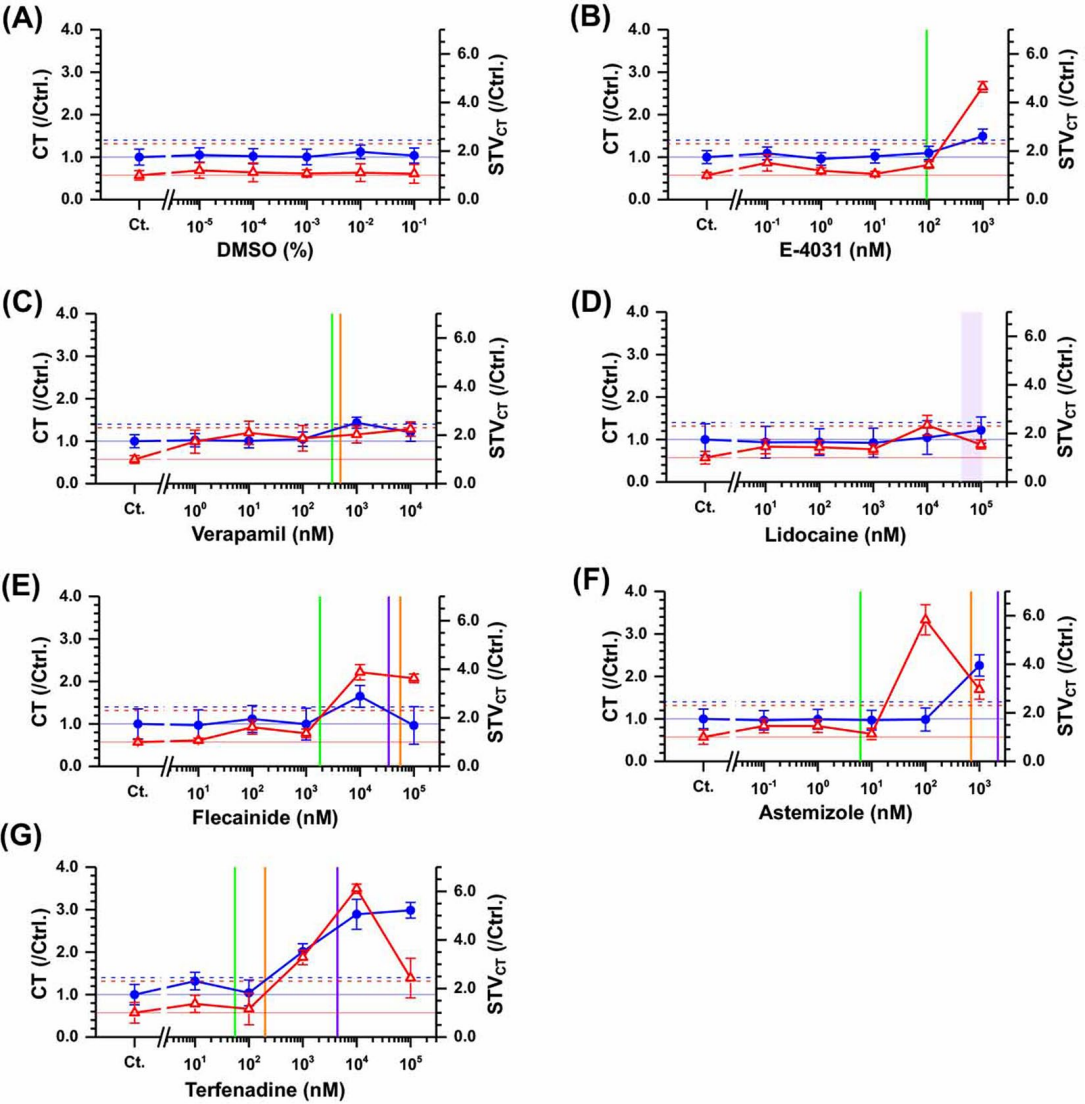
Effects of arrhythmic drugs on conductivity and its temporal fluctuation in the line-shaped model using hESC-CMs. Dose-dependent changes in the conduction time (CT) between two neighboring electrodes with a 150 *μ*m of inter-electrode distance and its short-term variability (STV_*CT*_, red open triangles and lines) are plotted for DMSO as a vehicle control (**A**), E-4031 as a I_*Kr*_ blocker (**B**), verapamil as a Cav1.2 blocker (**C**), lidocine and flecainide as I_*Na*_ blockers (**D** and **E**), astemizole and terfenadine as multi-ion channel blockers (**F** and **G**). CT and STV_*CT*_ were normalized to control condition. Symbols and error bars indicate mean values and standard error (SE). A 40% CT increase (CT > 1.4) and 130% STV_*cFPD*_ increase (STV_*cFPD*_ > 2.3) are shown iby blue and red dashed lines, respectively. The green, orange, and purple bars show hERG, Cav1.2, and Nav1.5 IC_50_ of each drug, respectively.

First, we evaluated the CT between two neighboring electrodes with 150 *μ*m of inter-electrode distance and its STV (STV_*CT*_) as the index of spatiotemporal fluctuation. As expected, CT did not significantly change at any concentration by pure hERG blocker E-4031 or Ca_2 +_ and hERG blocker, verapamil (Fig. 5B,C), which was similar to the results of DMSO as the vehicle control (Fig. 5A). However, STV_*CT*_ was increased by the 336% in comparison with the control condition (n = 13, P < 0.05) at 1 *μ*M of E-4031 (red symbols in Fig. 5B) and by 103% at 1 *μ*M of verapamil (red symbols in Fig. 5C).

Next, we examined two different types of sodium channel blockers, flecainide (class Ic) and lidocaine (class Ib), which are known to increase conduction time strongly or weakly, respectively^28^. Flecainide increased CT by 64% at 10 *μ*M (n = 4, P = 0.66, Fig. 5E), In contrast, lidocaine caused slight increase in CT at 100 *μ*M (22.1%, n = 4, P = 0.99, Fig. 5D). It appeared that both concentrations of these drugs would be sufficient to inhibit the I_*Na*_ current, because the half maximal inhibitory concentrations (IC_50_) of Nav1.5 by flecainide and lidocaine were 34 and 42–108 *μ*M, respectively, in consideration of roughly the same plasma protein-binding rate (60–80%, Table 1). STV_*CT*_ was increased by 10 *μ*M flecainide (287%, P = 0.76, Fig. 5E). However, no significant increase were found at 10 or 100 *μ*M lidocaine (134%, P = 0.99, 54%, P = 1.00, respectively, Fig. 5D).

**Table 1:** Effects of ion channel blockers on cFPD, STV_*cFPD*_, conduction time (CT), STV_*CT*_, inter-spike interval (ISI) and beat rate.

Drug Unbound (nM)^8,55^ IC_50_^10^ (nM)	Conc. (nM)	n	cFPD (ms)	STV_*cFPD*_ (ms)	Cond. Time (ms)	STV_*CT*_ (ms)	ISI (s)	BR (bpm)	Arrest	EPTC^8^ (nM)
DMSO n.d. n.d.	Ctrl.	9	288 ± 28	50 ± 31	1.2 ± 0.7	0.57 ± 0.33	0.76 ± 0.04	79 ± 4		n.d.
	0.1%	9	−0.51%	−6.7%	4.9%	20%	−3.5%	3.6%		
	0.2%	9	0.77%	−1.2%	2.2%	12%	−4.8%	5.0%		
	0.3%	9	0.75%	7.7%	0.38%	7.5%	−5.1%	5.4%		
	0.4%	9	1.1%	2.3%	12%	11%	−5.2%	5.6%		
	0.5%	9	−6.0%	18%	3.7%	6.2%	−7.5%	8.3%		
Astemizole 0.2–0.26 hERG: 6.2 Nav1.5: 2,200 Cav: 700	Ctrl.	5	250 ± 47	38 ± 30	1.4 ± 0.7	0.62 ± 0.41	0.75 ± 0.06	80 ± 6		5.5–7.9
	10^−1^	5	−0.71%	−2.8%	−3.3%	46%	−1.9%	2.0%		
	1	5	0.24%	−14%	−0.99%	46%	−5.4%	6.0%		
	10	5	0.69%	3.5%	−3.0%	13%	−5.9%	6.5%		
	10^2^	5	4.4%	14%	−1.4%	483%	−5.3%	6.1%		
	10^3^	3	44%	122%	126%	196%	3.1%	−2.5%	2/8	
E-4031 1.09 hERG: 91 Nav1.5: >1,000 Cav1.2: n.d.	Ctrl.	13	266 ± 41	58 ± 59	1.6 ± 0.9	0.49 ± 0.21	0.79 ± 0.11	78 ± 9		3.5
	10^−1^	13	0.52%	2.9%	8.9%	52%	−2.5%	2.6%		
	1	13	0.69%	−3.6%	−4.3%	19%	−3.7%	3.9%		
	10	13	7.1%	17%	2.1%	5.7%	−4.2%	4.5%		
	10^2^	13	30%	94%	10%	43%	−4.7%	5.1%		
	10^3^	12	47%	110%	49%	3364%	−5.1%	5.8%	1/13	
Flecainide 278 hERG: 1,800 Nav1.5: 34,000 Cav1.2: 55,900	Ctrl.	4	223 ± 30	31 ± 22	0.7 ± 0.5	0.37 ± 0.08	1.03 ± 0.10	58 ± 5		483–2,413^66^
	10	4	0.13%	1,4%	−3.2%	7.0%	−2.5%	2.6%		
	10^2^	4	3.4%	−16%	12%	61%	−3.7%	3.9%		
	10^3^	4	1.8%	0.32%	−0.65%	36%	−4.2%	4.5%		
	10^4^	4	0.11%	41%	65%	288%	−4.7%	5.1%		
	10^5^	3	−14%	54%	−3.6%	263%	−5.1%	5.8%	1/4	
Lidocaine 1,804–7,732^67^ hERG: n.d. Nav1.5: 42,000–108,000^68^ Cav1.2: n.d.	Ctrl.	4	250 ± 54	24 ± 11	1.0 ± 0.77	0.73 ± 0.38	1.03 ± 0.17	60 ± 12		5,970–25,603^69^
	10	4	9.5%	54%	−6,4%	46%	−3.4%	3.6%		
	10^2^	4	10%	38%	−6.0%	43%	−4.2%	4.4%		
	10^3^	4	9.7%	54%	−7.4%	35%	−2.6%	2.8%		
	10^4^	4	−18%	345%	4.7%	134%	128%	−48%		
	10^5^	4	−18%	143%	22%	54%	160%	−51%		
Terfenadine 0.1–0.29 hERG: 55 Nav1.5: 4,400 Cav1.2: 200	Ctrl.	7	273 ± 34	58 ± 41	1.7 ± 1.1	1.3 ± 1.5	0.80 ± 0.12	76 ± 9		3.3–9.7
	10	7	2.3%	76%	32%	37%	−4.0%	4.6%		
	10^2^	7	2.8%	−3.4%	4.0%	16%	−2.0%	2.0%		
	10^3^	7	0.25%	37%	101%	228%	3.1%	−3.0%		
	10^4^	4	−26%	65%	189%	511%	122%	−38%	3/7	
	10^5^	2	−73%	243%	198%	143%	744%	−88%	2/4	
Verapamil 25–81 hERG: 350 Nav1.5: 66,800 Cav1.2: 500	Ctrl.	4	251 ± 37	29 ± 16	0.9 ± 0.3	0.36 ± 0.11	0.91 ± 0.12	250–810 ± 9		250–810
	1	4	−1.1%	14%	2.4%	73%	−0.97%	1.2%		
	10	4	−0.74%	−22%	0.86%	109%	−3.4%	3.9%		
	10^2^	4	−1.5%	−33%	4.8%	87%	−4.3%	4.7%		
	10^3^	4	0.10%	81%	43%	103%	−16%	30%		
	10^4^	3	−27%	−47%	21%	125%	−21%	26%	1/4	

Astemizole increased CT by 126% at 1 *μ*M (n = 3, P < 0.05, Fig. 5F) and terfenadine also increased CT by 189% at 3 *μ*M (n = 4, P < 0.05, Fig. 5G). IC_50_ of Nav1.5 by astemizole and terfenadine was 2.2 and 4.4 *μ*M, respectively (Table 1). STV_*CT*_ was increased by 0.1 and 1 *μ*M of astemizole (483% or 196%, P = 0.16 or P = 0.92 respectively, Fig. 5F) as well as 1 *μ*M, intermediate concentration of terfenadine (228%, P = 0.12, Fig. 5G), and higher concentrations.

### Risk assessment of arrhythmogenecity

**Figure 6:**
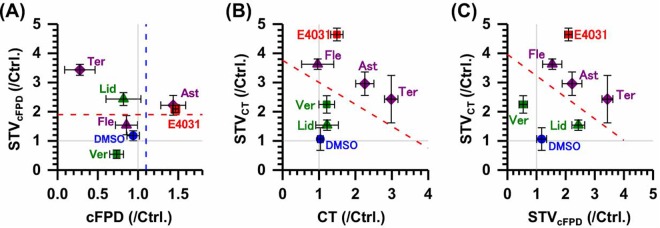
Summary of the effects of overdosed cardiotoxic compounds on cFPD, CT, and their STV of hESC-CMs. cFPD vs. STV_*cFPD*_ (**A**), CT vs. STV_*CT*_ (**B**) and STV_*cFPD*_ vs. STV_*CT*_ (**C**). All parameters were normalized to the control condition before addition of drugs. The concentration of the compounds were higher than therapeutic C_*max*_. E-4031; 1 *μ*M, astemizole (Ast): 1 *μ*M, flecainide (Fle): 10^2^ *μ*M, terfenadine (Ter): 10 *μ*M, lldocaine (Lid): 10^2^ *μ*M, verapamil (Ver): 10 *μ*M. Red symbols: positive compounds; purple symbols: false negative compounds; green symbols: negative compounds; blue symbols: vehicle. Square symbols: I_*Kr*_, or I_*Ca*_ blocker; triangle symbols: I_*Na*_ blockers; diamond symbols: multi-ion channel blockers. Red dashed line in (**A**) shows a 1.9 risk value in the cluster model shown by Kaneko *et al*.^40^. The red dashed lines in (**B** and **C**) were determined by the maximum differences from the line to the nearest point in each negative and positive compound data (equal) (Excel 2013 solver).

In Table 1, all drugs used in the MEA assay employing lined-up hESC-CMs are summarized with arrhythmogenic markers (cFPD and STV_*cFPD*_) and other parameters (CT, STV_*CT*_, Inter-spike interval, first peak amplitude, and arrest case). A cFPD and STV_*cFPD*_ plot at the highest dose of each drug did not discriminate non-arrhythmic lidocaine from pro-arrhythmic drugs (E-4031, astemizole, terfenadine, and flecainide) (Fig. 6A). In contrast, a CT and STV_*CT*_ or a plot of STV_*CT*_ and STV_*cFPD*_ could clearly discriminate between arrhythmogenic drugs (E-4031, flecainide, astemizole and terfenadine) and safe drugs (lidocaine and verapamil) clearly (red dashed line in Fig. 6B,C).

## Discussion

The electrophysiological analysis of hPSC-CMs is currently the most ideal strategy for *in vitro* QT/arrhythmogenic risk assessment. In particular, FPD assessment in the MEA assay using hPSC-CMs shows the potential to predict the QT/arrhythmogenic risk with much better reliability compared with other conventional *in vitro* assays^6–8,10,49,50^, although only FPD prolongation cannot fully predict lethal arrhythmia especially for evaluation of drugs with multiple ion channel effects^26^. Either spatial heterogeneity or temporal fluctuation would lead VT and lethal arrhythmia^51–53^. Regarding the temporal fluctuation, not only the conventional STV_*FPD*_ or STV_*cFPD*_ analysis but also the analysis including the individual or experimental differences Pueyo2016 or other cardiovascular related parameters, such as left ventricular pressure in *in vivo*^54^ have been developed for the TdP risk related parameters. However, there is no report that simultaneously measures the spatial fluctuation and temporal fluctuation in hPSC-CMs, and little is known about the combination potential of those indexes on arrhythmogenic risk assessment. The MEA assay has the advantage of observing multipoints in the cell community. Nevertheless, there are poor insights into the spatial fluctuation of conduction in the MEA assay. In the present study, we showed that not only the temporal fluctuation of cFPD, but also the spatial fluctuation of conduction time (CT) of the lined-up hPSC-CMs in the MEA assay can be additional indices as proarrhythmic markers. Our results indicated that the lined-up hESC-CMs can be used to evaluate cell-to cell conduction in combination with QT prolongation as well as discriminate between arrhythmogenic and safe drugs with multiple ion channel effects (Fig. 6A–C) by spatiotemporal fluctuation parameters, STV_*cFPD*_ and STV_*CT*_, or CT and STV_*CT*_.

In this study, our lined-up hESC-CMs provided reliable FPD response similar to conventional two-dimensional (2D) cell sheets exposed to well-known hERG channel blocker E-4031 as an FPD prolonger. FPD prolongation by E-4031 was demonstrated at the relevant concentration to the IC_50_ of hERG. Calcium channel blocker verapamil as an FPD-shortening drug also showed reasonable responses similar to the previously reported cFPD-shortening concentration response in 2D cell sheet or clusters of hPSC-CMs at 1–10 *μ*M^8,40^. However, the effects of astemizole on cFPD were seen at relatively higher concentrations compared with previous studies, especially for astemizole^6^, and at least about a 160 times higher concentration to the IC_50_ of hERG.

Regarding to the discrepancy of the drug response in cFPD among our results and previous reports using the MEA assay with hPSC-CMs, the difference of cell origins (hESC-CMs vs. hiPSC-CMs), cell arrangements (lined-up, vs. 2D cell sheet), cell density (274 ± 59 cells/mm with 60 *μ*m width in the lined-up system), and the duration of drug exposure, might be related, although there is no obvious explanation for this difference.

Furthermore, the present assay conditions did not detect cFPD prolongation of terfenadine or flecainide, which is in contrast to previous studies^5,55^. Terfenadine showed FPD shortening at concentrations more than 10 *μ*M (180 times higher than its hERG IC_50_) in this study. In addition, flecainide did not show FPD prolongation at concentrations of up to 100 *μ*M (55 times higher than its hERG IC_50_). It has been shown that drugs with a CaV1.2 inhibition potential have a counter effect on FPD prolongation by their hERG inhibition. IC_50_ of Cav1.2 can explain our observation because it is approximately four times higher than the IC_50_s of hERG for flecainide and terfenadine. In addition, terfenadine shows a bell-shape change in cFPD prolongation^5,55^, which cause difficult in evaluating its effect on cFPD. In fact, some reports indicate that terfenadine prolongs cFPD^4,7,8,49^ whereas others reported that terfenadine had little effect on cFPD^50^. Terfenadine is also reported to be one of the difficult drugs to detect the arrhythmogenicity of within a short period of exposure^56–59^, and this can be detected only after longer exposure of more than 12 h^60^. The time-course analysis would be required to detect arrhythmogenicity more precisely.

To investigate the CT and variability of CT (STV_*CT*_), we focused on two types of well-known sodium channel blocker, flecainide (class Ic) with a slowdown effect on the conduction velocity and lidocaine (class Ib) with little such effect. From the results, flecainide tended to increase CT and STV_*CT*_ at the second highest concentration, which is approximately equivalent to the therapeutic concentration, whereas lidocaine had a lesser effect than flecainide even at the highest concentration that is approximately 10 times higher than the therapeutic concentration (Fig. 5D,E). In addition, multi-ion channel blockers astemizole and terfenadine showed marked increases in CT and STV_*CT*_ at their equivalent concentrations to IC_50_s of Nav1.5 (Fig. 5F,G and Table 1). All drugs with Nav1.5 inhibition showed increases in CT at almost the same concentration as their IC_50*s*_ of Nav1.5, suggesting that CT, a parameter of cell-to cell conduction, would be relevant to Nav1.5 inhibition. This finding is well concordant with the recent report using the 2D-sheet of iPS-CMs^61^.

Moreover, Izumi-Nakaseko and colleagues stated that conduction properties of the cell sheet may largely depend on the extent of Nav1.5 availability as is the case in the human ventricle. Interestingly, a plot of CT and STV_*CT*_ discriminated between torsadogenic and non-torsadogenic compounds. Nevertheless, a plot of FPD and STV_*FPD*_ resulted in a false-positive judgment as arrhythmogenic for lidocaine in the lined-up cell network model (Fig. 6).

E-4031 and verapamil did not increase the mean value of CT at any tested concentration. However, both drugs increased the temporal beat-to-beat fluctuation, that is, STV_*CT*_ at the highest concentration of E-4031 or from the lowest concentration of verapamil (Table 1). It appears that the increase in STV_*CT*_ has no relationship to Nav1.5 inhibition, because IC_50_s of Nav1.5 in both E-4031 and verapamil are higher than their highest tested concentrations in this study.

Quality and quantity control of cardiomyocyte network should also be considered for establishment of reproducible on-chip *in vitro* assays. The influence of fibroblasts has been examined in on-chip cell-network assay, indicating that fibroblasts reduce the ability of synchronization of cardiomyocytes^62,63^. Heterogeneity of cardiomyocytes also should be a issue for improvement. We have previously evaluated the single cells obtained from single hESC-CMCs visually and concluded that 73% of total cells beat spontaneously, however their action potential analysis of APD_20–40_/APD_50–70_ and dV/dt_*max*_ revealed that they would have at least more than two major phenotypes, arbitrary “ventricular-like” and “atrial-like” cells^64^. To overcome this problem of mixture of phenotypes, computational simulation might be one of the possible solutions. For example, the role of community effect of single phenotype cardiomyocyte network was simulated successfully with fluctuation-dissipation theorem^65^.

As a limitation of this study, the following four aspects should be noted. We used only one positive (flecainide) and one negative (lidocaine) control for conduction-slowing effect. Further study using diverse blockers, such as other types of sodium channel blockers and gap junction inhibitors, would deepen the understanding of each parameter recorded in our MEA assays. Second, because we cannot deny the possibility of drug-induced chronotropic effect on CT evaluation, pacing would be helpful to eliminate chronotropic effect, which would enable us to evaluate the drug effects on CT directly. Third, a comparison of the expression balance of each cardiac ion channel in each cell arrangement model (lined-up, clusters and 2D cell sheets) is also important, because there are differences in response in cFPD prolongation among those assays^64^. Finally, we need to consider the importance of community effects of cardiomyocytes, such as the cell number, spatial arrangement, and cell contamination by non-cardiomyocytes.

In conclusion, the lined-up cell-network model of the MEA assay using hESC-CMs allows small scale *in vitro* simultaneous evaluation of FPD and CT, that is, two important indexes of lethal arrhythmia occurrence, repolarization and depolarization. STV_*FPD*_ and STV_*CT*_ also discriminated between torsadogenic and non-torsadogenic compounds with significant increases in both of their fluctuation values, indicating they are potential new indexes to predict torsadogenic lethal arrhythmia risk more precisely.
